# Evaluation of ceftriaxone use in the medical ward of Halibet National Referral and teaching hospital in 2017 in Asmara, Eritrea: a cross sectional retrospective study

**DOI:** 10.1186/s12879-019-4087-z

**Published:** 2019-05-24

**Authors:** Yohana Haile Berhe, Nebyu Daniel Amaha, Amon Solomon Ghebrenegus

**Affiliations:** 1 Pharmacy, Halibet National Referral and teaching Hospital, Asmara, Eritrea; 2 Department of Pharmacology, School of Pharmacy, Asmara College of Health Sciences, Asmara, Eritrea; 3 Ghindae Zonal Referral Hospital, Ghindae, Eritrea

**Keywords:** Eritrea, Ceftriaxone, Medical ward, Halibet national referral and teaching hospital

## Abstract

**Background:**

Antibiotic resistance due to overuse of antimicrobials is an issue that has been of concern to many health institutions and society in general. Resistant infections have high impact in low income countries since they can’t afford more recent and expensive antibiotics. Studies that evaluate antibiotic use in hospitals are scarce in Eritrea. Ceftriaxone is commonly available in Halibet National Referral and teaching hospital (HNRTH). Resistance to this antibiotic would have a great impact on the hospital since there is no other available third generation cephalosporin or higher classes of antibiotics.

**Method:**

A retrospective cross sectional design was used to evaluate the use of ceftriaxone in patients admitted to the medical ward in 2017. Clinical card number of inpatients who took ceftriaxone was extracted from the database of the Satellite Pharmacy Department of HNRTH and collected using a standardized data collection form. A descriptive analysis was employed and the Statistical package for social sciences (SPSS), version 20 was used for analysis.

**Results:**

A total of 120 patients were taking ceftriaxone for various indications. There were 55 (50.5%) males and 54 (49.5%) females. 59.8% of the patients were treated in the range of 0–7 days. The mean age was 56 (SD: 20.7). On average patients were under treatment for 6 days. The proportion of patients taking ceftriaxone was 11.43% out of all admissions in the medical ward. One, two or three antibiotics were co-prescribed with ceftriaxone in 39.4%. The most commonly co-prescribed antibiotic was gentamycin, accounting for 16.4% of the co-administered antibiotics. The most common indications for ceftriaxone were pneumonia, sepsis, TB, and CHF. Ceftriaxone therapy was appropriate in 30 (27.5%) cases and 68 (62.4%) cases were inappropriate in any of the four parameters of assessment used.

**Conclusion:**

Inappropriate use of ceftriaxone was found to be high in the hospital. This calls for establishment of hospital and national guidelines of antimicrobial treatment. Moreover drug restriction and antibiotic stewardship implementation in the hospital should be sought to prolong the lives of important drugs like ceftriaxone.

## Background

Overuse of antimicrobials and the resulting resistance is one of the issues threatening global public health [1–3]. It has been noted that infections caused by bacteria resistant to a specific antibacterial drug consume more health care resources and are associated with an increased risk of worse clinical outcomes and death [4]. Low income countries are heavily affected by antibiotic resistance due to high rate of infectious diseases and fewer antibiotic options [5]. Due to increasing resistance, cephalosporins are frequently identified as a particular target for use evaluation and antibiotic stewardship [6].

Ceftriaxone is one of the third generation cephalosporins that is commonly prescribed due to its low toxicity and high efficacy against a wide range of bacteria [7–9]. However, in an antimicrobial surveillance done by the WHO on all its six regions, the resistance pattern of *Escherichia coli (E. coli)* to third generation cephalosporins was 50% or more in five regions. All six regions reported *Klebsiella pneumonia* (*K. pneumonia*) had developed resistance in 50% or more cases while three regions reported that *Neisseria gonorrhoea* had developed 25% or more resistance to third generation cephalosporins [4].

Moreover ceftriaxone use is linked with vancomycin resistant enterococci and penicillin resistant streptococci [10–12]. The emergence of extended-spectrum beta-lactamases, associated with overuse of third generation cephalosporins, has led to treatment failures in infections of certain bacteria like the Enterobacter [13] and Enterobacteriacae species [14–16].

Understanding the relationship between emerging bacterial resistance and antibiotic use requires monitoring antibiotics both locally and nationally [17]. Ward level antibiotic use studies generate detailed information about antibiotic consumption and rationality of prescribing [18].

In Eritrea, there is a paucity of research done on antibiotic use in hospitals and no studies have been done on ceftriaxone use. Ceftriaxone is the most potent antibiotic available in Halibet National Referral and teaching hospital (HNRTH). There are no other available third generation cephalosporins or higher classes of antibiotics in the hospital, thus resistance to this antibiotic would have a significant impact. In light of this fact it seems prudent to use ceftriaxone responsibly. It is by evaluating its use and prescription habits that such prudence can be translated into policy and restriction in its use and stewardship implemented.

The aim of this study was to evaluate drug use patterns of ceftriaxone and assess appropriateness of therapy. All patients admitted to the medical ward in 2017 and who took ceftriaxone were included in the study. The findings of study will help in identifying improper aspects of ceftriaxone use.

## Methods

### Study setting

HNRTH is one of the major referral hospitals in Eritrea located in the capital city, Asmara. It is a 180 bed national referral and teaching hospital with surgical, medical, orthopaedic, burn and emergency wards. The hospital also provides out-patient services. HNRTH received around 6000 admissions in 2017. The study was done in the medical ward which has one wing for females with 15 beds and another wing for males with 24 beds.

### Study design

A retrospective cross sectional design was used to evaluate the use of ceftriaxone in patients admitted to the medical wards from January 1 to December 31 of 2017.

### Inclusion and exclusion criteria

All patients admitted to the medical ward of HNRTH from January 1 to December 31 of 2017 and who took at least one dose of ceftriaxone were included in the study. Those patients whose clinical card couldn’t be located from the records office were excluded.

### Data collection form

Data was collected using a structured format with the necessary fields.

### Data collection procedure

Clinical card numbers of all patients admitted to the medical ward and who took at least one dose of ceftriaxone were extracted from the database of the Satellite Pharmacy Department of HNRTH. The clinical card numbers were used to retrieve the clinical records of each patient. Data pertaining to the research was filled from the records using a standardized data collection forms designed for the research.

### Ethical approval

Administrative permission was required for accessing raw data used in the study. This permission was granted by the medical director office of Halibet National Referral and teaching hospital, which is responsible for ethical approval of researches done in the hospital. The hospital doesn’t have a formal ethics review committee and all ethical reviews of researches conducted in the hospital are done by the medical director office. The confidentiality of the data collected was maintained and all patient identifiers were removed.

### Data analysis

The collected data were checked for completeness and entered in to the Statistical Package for Social Sciences (SPSS), version 20 for analysis. The indication, dose, frequency and duration were used as measurements of ceftriaxone appropriateness according to WHO drug use evaluation parameters. Other parameters, like drug-drug interaction and sensitivity testing were not included as parameters because our set up doesn’t have the necessary equipment for analysis. Final diagnosis upon discharge was used for assessment of indication of ceftriaxone. The Ethiopian standard treatment guidelines for general hospitals of 2014 was used [19] because there were no up to date national or hospital standard treatment guidelines of Eritrea at the time of the study. These two countries are neighbouring countries with many similarities in the health care system, thus authors believe comparison is valid. Inappropriate use of ceftriaxone in the hospital was determined by comparing the observed ceftriaxone prescribing in the chart records to the recommendations in the guideline. A descriptive analysis was employed and results are presented in tables and a figure.

## Results

A total of 1049 patients were admitted to the medical ward in the period under study; out of those 120 patients were taking ceftriaxone for various indications. The clinical records for 11 cases were missing and only 109 were evaluable. There were 55 (50.5%) males and 54 (49.5%) females (Table 1). Majority of the treatment duration was in the range of 0–7 days accounting for 59.8% (Table 1). The mean age was 56 years (SD: 20.7). On average patients were under treatment for 6 days (SD: 3.82). In 94.5%, ceftriaxone was administered as a 2 g daily dose. The proportion of patients taking ceftriaxone was 11.43% out of all admissions in the medical ward. A chi-square test was performed to find if there was an association between age, gender and appropriateness of treatment of the cases. There was no significant relationship found between these categories.

**Table 1:** Age, gender distribution and duration of treatment of ceftriaxone in medical wards of HNRTH

		Count	%
Gender	Female	54	49.5
	Male	55	50.5
Age (years)	15–65 years	66	60.6
	> 65 years	43	39.4
Duration of treatment	0–7 days	64	59.8
	8–14 days	38	35.5
	> 14 days	5	4.7
	Total	109	100

### Most commonly co-prescribed antibiotics

Ceftriaxone was prescribed alone in 60.6% of the cases whereas the remaining 39.4% had either one, two or three antibiotics co-prescribed with it. The most commonly co-prescribed antibiotic was gentamycin, accounting for 16.4% of the co-administered antibiotics. Anti-tuberculosis drugs and ciprofloxacin were also prescribed often (12.7% each) (Table 2).

**Table 2:** Frequency of antibiotics co-prescribed with ceftriaxone

Drug name	N	%	% of cases
Gentamycin	9	16.4	20.9
Ciprofloxacin	7	12.7	16.3
Anti-tuberculosis drugs	7	12.7	16.3
Metronidazole	6	10.9	14.0
Ampicillin	4	7.3	9.3
Benzyl penicillin	4	7.3	9.3
Cloxacilline	4	7.3	9.3
Sulfamethaxazole+Trimethoprim	4	7.3	9.3
Amoxicilline+Clavulinic acid	3	5.5	7.0
Clindamycin	2	3.6	4.7
Artesunate	2	3.6	4.7
Artesunate+ Amodiaquine	1	1.8	2.3
Chloramphenicol	1	1.8	2.3
Quinine	1	1.8	2.3
Total	55	100	127.9

### Common indication for ceftriaxone use

The most common indications for ceftriaxone were pneumonia, sepsis, TB, and CHF in descending order. It was also prescribed for UTI, PUD, anemia, and stroke. (Table 3).

**Table 3:** Most common indication for ceftriaxone utilization

Diseases	Responses	
	N	%
Pneumonia	40	18.5
Sepsis	14	6.5
CHF	14	6.5
TB	14	6.5
Anemia	6	2.8
UTI	6	2.8
PUD	5	2.3
Stroke	5	2.3
RVI	4	1.9
Lung cancer	3	1.4
Meningitis	3	1.4
Hepatoma	3	1.4
CML	3	1.4
parapneumonic effusion	3	1.4
Abscess	2	0.9
CLD	2	0.9
Lung fibrosis	2	0.9
CRF	2	0.9
Ascites	2	0.9

Key: *CHF* Congestive Heart Failure, *TB* Tuberculosis, *UTI* Urinary tract infection, *PUD* Peptic ulcer disease, *RVI* Retroviral infection, *CML* Chronic myeloid leukaemia, *CLD* Chronic liver disease, *CRF* Chronic renal failure

### Overall appropriateness of ceftriaxone therapy

**Fig. 1:**
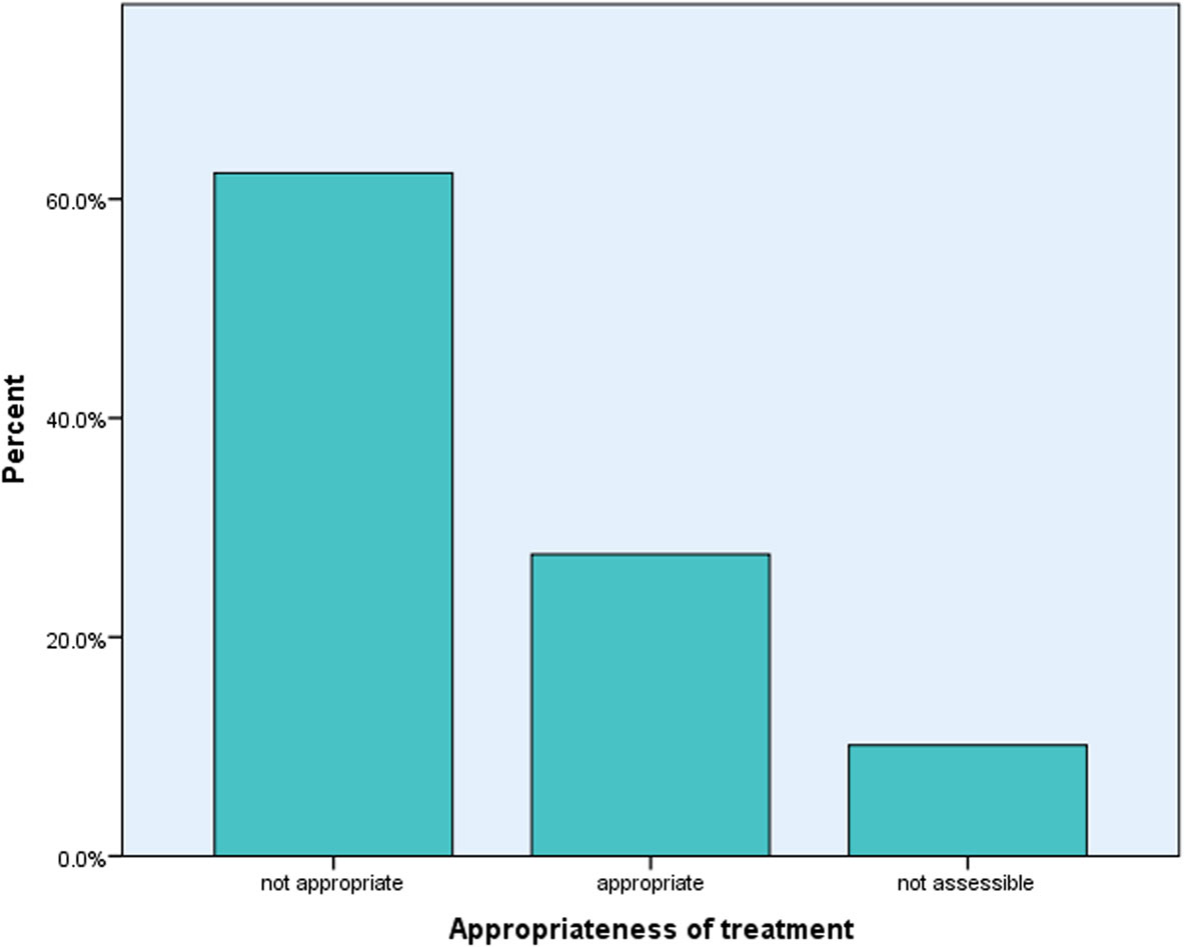
Appropriateness of ceftriaxone therapy

In 30 (27.5%) cases ceftriaxone therapy was appropriate in all the four parameters used; indication, dose, frequency and duration. While 68 (62.4%) cases were found to be inappropriate in any one of the four parameters of assessment (Fig. 1). However, 11 (10.1%) of the cases were not assessable due to incomplete information in either the diagnosis or the duration of treatment. Moreover, when data was analysed for appropriateness without accounting for duration of treatment; the number of appropriate cases increased to 55 (50.5%) with only 43 (39.4%) inappropriate. Therefore majority of the inappropriate use of ceftriaxone was seen in the duration of treatment (Table 4).

**Table 4:** Appropriateness of ceftriaxone use using four indicators of appropriate use

	Appropriately used		Inappropriately used	
	Frequency	%	Frequency	%
Right indication	56	51.4	42	38.5
Right dose	55	50.5	43	39.4
Right frequency	56	51.4	42	38.5
Right duration	29	26.6	68	62.4

Use was termed appropriate when all the four parameters were fulfilled and inappropriate when any one of the four parameters were not fulfilled.

## Discussion

This study was designed to assess the appropriateness of ceftriaxone prescription in the medical ward of HNRTH. Out of all admissions to the medical ward, 11.4% of the patients were administered at least one dose of ceftriaxone. This was much lower than studies in Ethiopia that reported a higher percentages ranging from 49 to 59.3% [3, 20, 21]. A study done in India also had higher incidence of ceftriaxone use at 48.5% [22], while a Tehran study reported ceftriaxone use at 34% [23]. Pereira et al. also found ceftriaxone to be the most common third-generation cephalosporin prescribed in the Port of Spain [7]. The lower prevalence of ceftriaxone use in our study might be owing to the inconsistency (e.g. stock outs of ceftriaxone in the hospital pharmacy) in availability of ceftriaxone.

In our study, we found the average duration of treatment to be 6.79 days (SD: 3.82). This finding is consistent with studies done in Dessie, Ethiopia (6.7) [24] and Ayder, Ethiopia (7.2 days) [25]. However, other studies carried out in Ethiopia found much higher mean duration of treatment ranging from 9.2 days to 11.47 days [3, 21, 26]. Studies carried out in the South Korea and the Port of Spain also found higher average duration of treatment at 10.3 and 14 days respectively [7, 9].

### Most commonly co-prescribed antibiotics

In our study, the most commonly co-prescribed antibiotic was gentamycin (16.4%) followed by ciprofloxacin and anti-tuberculosis drugs (12.7% each), and metronidazole (10.9%) (Table 2). A study in Dessie, Ethiopia found anti-tuberculosis drugs and cloxacillin to be the most commonly co-prescribed antibiotics [24] while another study in Gondar, Ethiopia found vancomycin and doxycycline to be commonly co-administered with ceftriaxone [3]. Sileshi et al. found metronidazole and vancomycin as the most commonly prescribed antibiotics [20]. Studies in Ethiopia found that IV fluids were the most commonly co-administered medication [3, 21, 24]. Ceftriaxone co-administration with ringer lactate was high in studies carried out in Police and Black Lion Hospital, Ethiopia (44.48%) [26], Ayder referral hospital, Ethiopia (33.65%) [25] and Tikur Anbessa Hospital (6.7%) [20]. These findings showed a potential risk for drug-drug interaction. In our study, we didn’t assess IV fluids because data on what type of fluid was used was unavailable and was mostly ordered as IV fluid.

### Common indication for ceftriaxone use

We found that the most common indications for ceftriaxone administration were pneumonia, sepsis, TB, and CHF (Table 3). The use of ceftriaxone for diseases like TB, PUD, CHF and anaemia was not warranted, however assessment was based on the final diagnosis recorded at the time of discharge and error in failing to record all infections patient has had may explain the administration of ceftriaxone for these illnesses. In addition patients with TB are more likely to present with co-morbidities since those patients are immunocompromised at the time of admission but negligence in recording patient history may introduce inaccuracy into the interpretation of our results. On the other hand empirical treatment is commonly employed in our setting, which depends heavily on the physicians’ clinical judgment and experience, therefore it is likely that some cases of non-infectious diseases may receive antibiotic treatment.

Many studies in Ethiopia came to the conclusion that RTIs are the most common indication for ceftriaxone therapy [3, 8, 21, 24–26]. All the above studies reported that UTI, CNS infections, sepsis and skin and soft tissue infections were among the most common indications for ceftriaxone therapy. This could be due to high infectious diseases rate in third word hospitals and high consumption of this drug in hospital admissions due to lack of alternative antibiotic options. A study done in Eritrea found that *Pseudomonas spp.* and *Klebsiella spp.* (some of the causative organisms of pneumonia) were resistant to ampicillin in 81.8 and 75% respectively [27]. Ampicillin is the most ubiquitous and easily available drug in the hospital. Therefore, these results suggest that there will be an increase in ceftriaxone use as there are no other accessible alternatives.

### Over all appropriateness of ceftriaxone therapy

Ceftriaxone therapy was inappropriate in either indication, dose, frequency or duration in 62.4% of the cases (Table 4). This was much higher than some studies carried out in Ethiopia which found ceftriaxone use to be inappropriate ranging from 46.2% [24] to 55.4% [21]. Other studies in Gondar and Tikur Anbessa hospital, Ethiopia [3, 20] as well as a study in Tehran [22], found much higher inappropriateness of use at 80.2, 87 and 85.3% respectively. A study done in South Korea reported inappropriate use of ceftriaxone at 34.5% only [9] but a study in the USA found it to be at 53% [28]. The low rate of ceftriaxone use in the South Korean study may be attributed to the fact that the study included culture and sensitivity test and other laboratory results as a parameters [9]. In Eritrea there is only one laboratory, the National Health Laboratory of Eritrea, which carries out culture and sensitivity tests. Moreover, it will take an average of 2 weeks for the laboratory results to be available, which could compromise the health status of the patient. Therefore, most of the physicians prefer treating the patient empirically than sending the culture and drug sensitivity test to laboratory.

In our study, most of the cases were inappropriate in the duration of treatment (Table 4). This was similar with findings in Ethiopia [21, 24–26]. Studies in Gondar and Tikur Anbessa reported frequency of administration as the most common cause for inappropriateness [3, 20] Durham et al. discovered lack of appropriate indication as the most common cause for inappropriateness [28].

Our study couldn’t find any association of inappropriate use of ceftriaxone with age and gender. This was consistent with a study in Tikur Anbessa Hospital, Ethiopia [20] but a study in Thailand found that there was a higher incidence of inappropriate use of ceftriaxone in females [29]. This may be attributed to the enrolment of a larger proportion of females in the Thailand study (60.8%) [29] compared to the present study (49.5%).

### Limitation of study

The retrospective nature of this project confers limitations inherent to this type of design. In particular, the determination of appropriateness was made by the authors based on the information provided through chart review. Thus, it is possible that some patients who were classified as having received ceftriaxone inappropriately may in actuality have received it appropriately and vice versa due to interpretation of data and rationale or lack of data and rationale. In addition treatment of ceftriaxone was assessed only as appropriate in the diseases situation; there may have been cases where ceftriaxone use was not the fist-line treatment but given as one in the cases; lack of availability of alternative antibiotics may force prescribers to use whatever antibiotic is readily available. Moreover assessment of appropriate indication was done using final diagnosis written at the time of discharge, error in recording all superimposed infections patient may have had may make the interpretation of our data inaccurate. Co-prescription of IV fluids with ceftriaxone was not assessed because data on what type of fluid was used was unavailable as it was mostly ordered as IV fluid.

## Conclusion

The inappropriate use of ceftriaxone was found to be high in the hospital. This calls for development of hospital and national guidelines of treatment. Some of the reasons for inappropriate use of antibiotics are unavailability of alternative antibiotics and inconsistency in drug supply. Ensuring continuous supply of drugs and increasing the availability of antibiotic options may help in decreasing inappropriate use of ceftriaxone and other antibiotics. Drug restriction and antibiotic stewardship are also interventions that have proven beneficial in extending the life of antibiotics. Their implementation in the hospital would cultivate rational prescription of restricted drugs and ensure appropriate use. Finally more studies to assess the appropriate use of antibiotics need to be undertaken nationally to study use of antibiotics and estimate the extent of their inappropriate use.

## Abbreviations

CHF: Congestive Heart Failure
CLD: Chronic liver disease
CML: Chronic myeloid leukaemia
CNS: Central nervous system
CRF: Chronic renal failure
HNRTH: Halibet National Referral and Teaching Hospital
IV: Intravenous
PUD: Peptic ulcer disease
RTI: Respiratory tract infection
RVI: Retroviral infection
TB: Tuberculosis
UTI: Urinary tract infection
WHO: World Health Organization
